# Elevated α/β ratio after hypofractionated radiotherapy correlated with DNA damage repairment in an experimental model of prostate cancer

**DOI:** 10.1093/jrr/rrae077

**Published:** 2024-10-28

**Authors:** Ming Cui, Yuexian Li, Ji Liu, Deyu Sun

**Affiliations:** Department of Abdominal·Osteomalacia Radiotherapy Cancer Hospital of Dalian University of Technology, No. 44, Xiaoheyan Road, Dadong District, Shenyang, Liaoning Province China, Shenyang, Liaoning 110042, P.R. China; Department of Abdominal·Osteomalacia Radiotherapy Cancer Hospital of Dalian University of Technology, No. 44, Xiaoheyan Road, Dadong District, Shenyang, Liaoning Province China, Shenyang, Liaoning 110042, P.R. China; Department of Abdominal·Osteomalacia Radiotherapy Cancer Hospital of Dalian University of Technology, No. 44, Xiaoheyan Road, Dadong District, Shenyang, Liaoning Province China, Shenyang, Liaoning 110042, P.R. China; Department of Abdominal·Osteomalacia Radiotherapy Cancer Hospital of Dalian University of Technology, No. 44, Xiaoheyan Road, Dadong District, Shenyang, Liaoning Province China, Shenyang, Liaoning 110042, P.R. China

**Keywords:** Α/β ratio, DNA damage, DNA repair, Γ-h2ax, Rad51

## Abstract

Our previous study demonstrated that the linear quadratic model appeared to be not well-suited for high dose per fraction due to an observed increase in α/β ratio as the dose per fraction increased. To further validate this conclusion, we draw the cell survival curve to calculate the α/β ratio by the clone formation experiment and then convert the fractionated radiation dose into an equivalent single hypofractionated radiation dose comparing with that on the survival curve. Western Blot and laser confocal immunofluorescence were used to detect the expression of γ-H2AX and RAD51 after different fractionated modes of radiation. We constructed a murine xenograft model, and changes in transplanted tumor volume were used to evaluate the biological effects after different fractionated radiation. The results demonstrated that when fractionated radiation dose was converted into equivalent single hypofractionated radiation dose, the effectiveness of hypofractionated radiation was overestimated. If a larger α/β ratio was used, the discrepancy tended to become smaller. γ-H2AX was higher in 24 h after a single high dose radiation than the continuous expression of the DNA repair marker RAD51. This implies more irreparable damage in a single high dose radiation compared with fractionated radiation. In the murine xenograft model, the effectiveness of hypofractionated radiation was also overestimated, and additional fractions of irradiation may be required. The conclusion is that after single hypofractionated radiation, the irreparable damage in cells increased (α value increased) and some repairable sublethal damage (β value) was converted into irreparable damage (α value). When α value increased and β value decreased, the ratio increased.

## INTRODUCTION

The main application of the α/β ratio in tumor radiobiology was to evaluate the sensitivity of tumors and surrounding tissues to radiation and then to guide the selection of radiotherapy [1]. According to the cell survival curve of the linear quadratic (LQ) model, α represents irreparable lethal damage and β represents reparable sublethal damage (SLD) [2–5]. Therefore, according to the definition of the α/β ratio, after DNA damage, if SLD repair occurs, the α/β ratio decreased (β value increased), the survival curve shifted upward and cells survival increased. When the repairment is not met in time, the α/β ratio increased (α value increased or β value decreased), and the survival curve shifted downward, resulting in decreased cell survival. This also explains why the α/β ratio of early reaction tissue is large and that of late reaction tissue is small.

Our previous study indicated that the α/β ratio was not a constant and increased with the increasing dose per fraction, and the LQ model, based on the view that the α/β ratio was a constant, may not be suitable for a single high-dose radiation [6]. To confirm this conclusion, we explored the mechanisms of the increased α/β ratio after hypofractionated radiation *in vitro* and *in vivo*.

DNA damage after ionizing radiation leads to DNA double-strand breaks (DSB). The expression of the phosphorylated histone H2A variant γ-H2AX, as a marker of DSB, is increased within a few minutes after irradiation and peaked within 30 min. γ-H2AX has a linear relationship with the amount of DNA double-strand damage. In other words, the expression of γ-H2AX increases with the increase of DSB and decreases with the repairment of DSB [7–9]. Therefore, the expression of γ-H2AX is a marker of DNA damage.

Homologous recombination (HR) is considered to be an error-free repair mechanism because it uses undamaged DNA double strands to guide repairment of broken DNA double strands and therefore is a high-fidelity repair approach. At the start of HR repair, ataxia telangiectasis mutated gene (ATM) is activated due to DSB, which phosphorylates the breast cancer 1 gene expressed protein (BRCA1), and is then recruited to the DSB site that has bound to the MRN protein complex (MRE11-RAD50s-NBS.1). BRCA2 is then recruited by BRCA1 to the DSB site, where it forms a complex with RAD51 to replace replicating protein A (RPA) to form nuclear protein filamentary. RAD51 is a key protein in HR that regulates the invasion of homologous sister chromatids and leads to the formation of Holliday junction. The Holliday junction is finally broken down into two DNA double-strands to complete HR repair [9–11].

Therefore, in this study, the expression levels of γ-H2AX (a DNA double-strand break damage marker) and RAD51 (a key protein in the HR repair pathway) were detected over time after different fractionated modes and observed the DNA damage repairment after hypofractionated radiation. In this case, according to the definition of the α/β ratio, we explore the reasons why the α/β ratio increased after hypofractionated radiation.

## MATERIALS AND METHODS

### Cell culture

Prostate cancer cell lines DU145 and PC3 (granted from the Department of Nuclear Medicine, Peking University First Hospital) were cultured in RPMI1640 medium containing 10% fetal bovine serum, 1% glutamine and 1% tri-antibodies (penicillin, streptomycin and amphotericin B). Incubator temperature is set at 37°C, CO_2_ concentration at 5% and an appropriate humidity is maintained. Cells in the logarithmic growth stage were taken for the experiment.

### Clone formation experiment

DU145 and PC3 cells were seeded in 6-well plates at a certain density, and 6 MV X-ray radiation was given ~12 h after the cells were stabilized and adhered to the plates. And 6-well plates were placed on the 1-cm-thick bolus because of the build-up region. X-rays were applied to a plate from below with source-surface distance (SSD) 100 cm. The irradiation doses were 0, 1, 2, 4, 6, 8, 12, 4 Gy/F × 2F and 6 Gy/F × 2F. The dose rate was 500 cGy/min. According to previous research reports, potentially lethal repair has been completed after 4 h [12, 13]. Considering that prostate cancer tissue is a late response tissue, the interval of radiation was extended to 6 h. Cells were cultured in an incubator until 14 days after radiation. The culture medium was changed ~6 days. The clone count was performed under the microscope. More than 50 cells are generally considered to be clones.

According to the clone formation rate at different radiation doses, the survival fraction of cells was calculated. Then, using the radiation dose (0, 1, 2, 4, 6, 8 and 12 Gy) as the *X*-axis, the survival fraction of cells at each radiation dose was used as the *Y*-axis to draw the cell survival curve using statistical software for curve fitting. Involved formulas were below:


$$ \mathrm{Plating}\ \mathrm{efficiency}\left(\mathrm{PE}\right)=\frac{\mathrm{Number}\ \mathrm{of}\ \mathrm{colonies}\ \mathrm{counted}}{\mathrm{Number}\ \mathrm{of}\ \mathrm{cells}\ \mathrm{seeded}}\times 100 $$

$$ \mathrm{Surviving}\ \mathrm{fraction}\left(\mathrm{SF}\right)=\frac{\mathrm{Irradition}\ \left(\mathrm{PE}\right)}{\mathrm{Control}\ \left(\mathrm{PE}\right)}. $$

For fractionated radiation (4 Gy/F × 2F and 6 Gy/F × 2F), the cell survival fraction was also obtained according to the clone formation, and the corresponding equivalent single hypofractionated radiation dose (actual value) was obtained according to the fitting curve comparing with the equivalent dose (calculated value) calculated by the biological effective dose (BED) formula (n_1_d_1_ (1+ d_1_ /α/β) = n_2_d_2_ (1+ d_2_ /α/β)). The difference between the calculated value and the actual value was used to prove the applicability of the BED formula in single hypofractionated radiation.

### Western blot

DU145 and PC3 cells in logarithmic growth phase were inoculated on a 10 cm petri dish. When the cells grew to ~80%, they were irradiated with 8 Gy/F × 1F and 4 Gy/F× 2F, and the interval of irradiation was 6 h. Total proteins were extracted 30 min, 6 h and 24 h after irradiation, respectively. The extracted total protein was quantified by the BCA method. The protein weight was 25 μg to each well, and 1× loading buffer was used to fill up to 10 μl for each well. Gel electrophoresis at constant voltage: Stacking gel: 80 V constant voltage 30 min; Separating gel: 120 V constant voltage 90 min. After gel electrophoresis, 200 mA constant current transfer for 2 h was performed to transfer the proteins on the gel to the membrane. After the transfer, the membrane was removed and placed in 5% nonfat dry milk (TBST dissolved) and blocked for 1 h. After blocking, the membrane was directly applied to the corresponding primary antibody (RAD51(Cell Signaling Technology, CST): 1:1000; γ-H2AX(CST): 1:1000; α-tubulin (Abcam) 1:7500) and incubated overnight at 4°C. On the second day, TBST was washed three times and the corresponding secondary antibodies (RAD51 and γ-H2AX: goat-anti-rabbit 1:1000; α-tubulin: goat-anti-mouse 1:500) were incubated for 1 h, and TBST was washed three times again for chemiluminescence.

### Laser confocal immunofluorescence

DU145 and PC3 cells in logarithmic growth phase were inoculated on special laser confocal petri dishes with 2 × 10^5^ cells in each dish. When the cells adhered to the wall and grew to ~80%, 8 Gy/F × 1F and 4 Gy/F × 2F were irradiated with an interval of 6 h. The following operations were performed after irradiation: 30 min, 6 h and 24 h [14]:

(1) Rinse with PBS for 3 min/f × 2f; (2) fixed: 4% paraformaldehyde (room temperature) for 20 min; (3) rinse with PBS for 5 min/f × 3f; (4) permeabilize: 0.5% Triton X-100 treatment (room temperature) for 20 min; (5) rinse with PBS for 5 min/f × 3f; (6) serum blocking: 1% bovine serum-PBST (containing 0.05% Tween20) was blocked at room temperature for 20 min; (7) incubated primary antibody: suck out and air-dried blocking buffers and then incubated with γH2AX (CST: 1:400) at 4°C overnight; (8) on the next day, rinse with PBST for 5 min/f × 3f; (9) incubated secondary antibody: the secondary antibody is fluorescent antibody (Alexa Fluor 488(CST): 1:1000), which is incubated at room temperature and avoid light for 1 h; (10) Rinse with PBST for 5 min/f × 3f; and (11) DAPI stained and anti-fluorescence quencher blocked: each well was blocked with anti-fluorescence quencher containing DAPI and then observed under a laser confocal microscope.

The above three experiments were repeated three times.

### Murine xenograft model

PC3 tumor cells were subcutaneously injected into the left hind legs of 6–8 week-old male nude mice. Tumor volume = (tumor length) × (tumor width)^2^/2. Mice were randomly divided into different fractionated groups (control, 2 Gy/f × 10f, 3 Gy/f × 5f, 3 Gy/f × 6f, 8 Gy/f × 1f and 8 Gy/f × 2f) and treated by local irradiation. Each group had six mice. Tumor volumes were measured twice weekly.

### Statistical analysis

SPSS 22.0 statistical software was used for statistical analysis. The data description of the measures was mainly expressed in the form of mean ± SD. Two-sample comparisons were performed by choosing *t*-test. Multiple comparisons were performed using two-way analysis of variance (ANOVA). Differences were considered statistically significant at *P* < 0.05.

## RESULTS

### If larger α/β ratio was used, the discrepancy between calculated value and actual value tended to become smaller

**Fig. 1:**
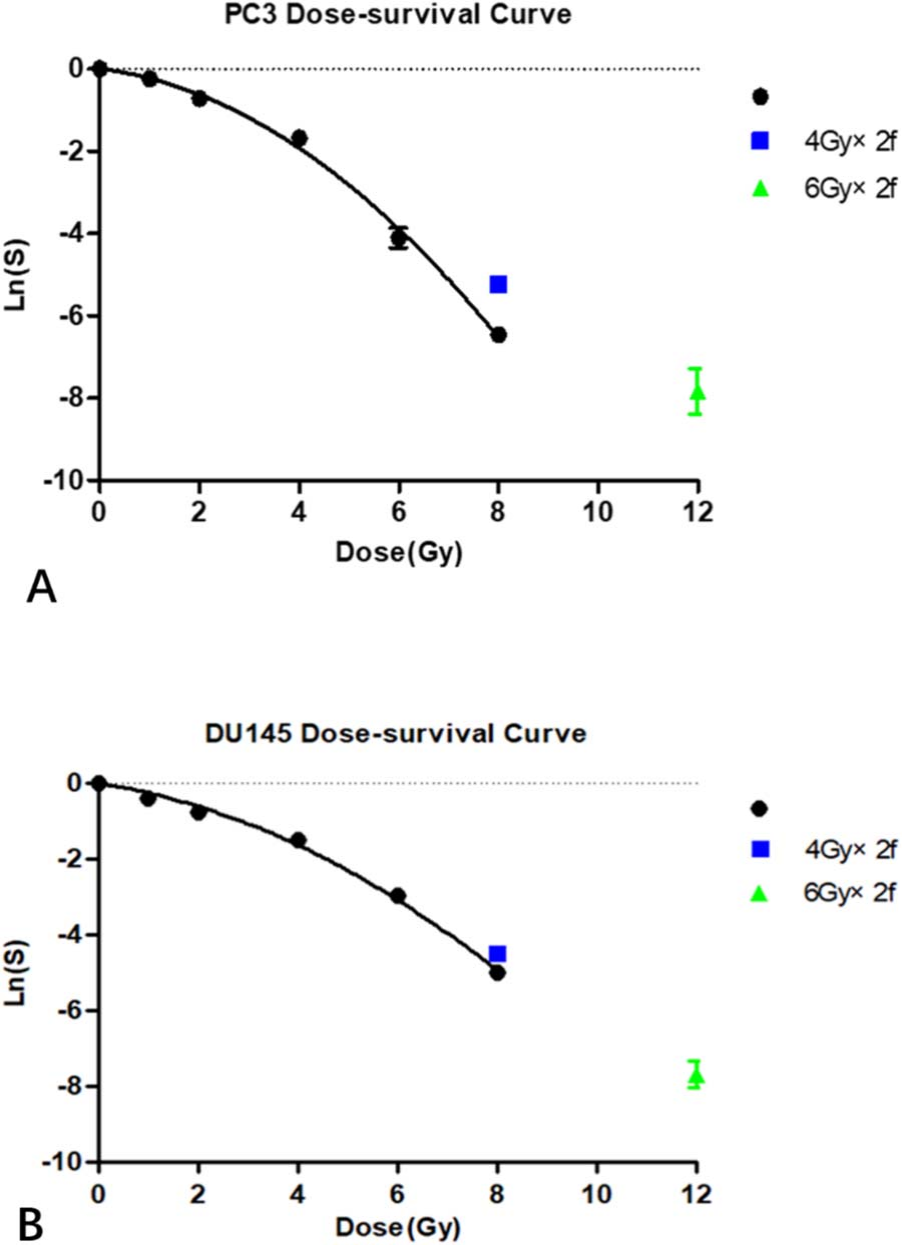
Dose-survival curves of PC3 cells (Fig. 1A) and DU145 cells (Fig. 1B). DU145 and PC3 cells were seeded on 6-well plates and 6 MV X-ray radiation was given after the cells were stabilized and adhered to the plates. The irradiation doses were 0, 1, 2, 4, 6, 8, 12, 4 Gy/F × 2F and 6 Gy/F × 2F. According to the clone formation rate at different radiation doses, the survival fraction of cells was calculated. Then, used the radiation dose as the *X*-axis, the survival fraction of cells at each radiation dose was used as the *Y*-axis to draw the cell survival curve of PC3 cells (Fig. 1A) and DU145 cells (Fig. 1B).

After the clone formation experiment, the survival fraction $S={e}^{-\alpha \cdotp d-\beta \cdotp{d}^2}$ can be obtained, and then, take the logarithm for both sides: $\ln (s)=-\alpha \cdotp d-\beta \cdotp{d}^2$and the survival curves of PC3 and DU145 were drawn as Fig. 1A and B**.**

The survival curve fitting results were as follows: ① PC3: α was 0.1461(95%CI: 0.04089–0.2514), β was 0.08366(95%CI: 0.06830–0.09902), α/β = 1.75 Gy, *R*^2^ = 0.9913 and the fitting equation was $\ln (s)=-0.15\cdotp d-0.08\cdotp{d}^2$. The survival fractions of 4 Gy/F × 2F and 6 Gy/F × 2F of fractionated radiation were corresponding to the curve and obtained corresponding single dose d_4 × 2_ = 7.19 Gy (actual value) and d_6 × 2_ = 9.16 Gy (actual value). At the same time, the α/β = 1.75 Gy was substituted into the BED formula: n_1_d_1_ (1 + d_1_/α/β) = n_2_d_2_ (1 + d_2_/α/β). In this formula, n_1_ value was 2; d_1_ values were 4 and 6 Gy, respectively; n_2_ value was 1 and we can calculate the equivalent single dose d_4 × 2_ = 5.96 Gy (calculated value) and d_6 × 2_ = 8.81 Gy (calculated value) according to the BED formula. (2) DU145: α was 0.1981 (95%CI: 0.1381–0.2581), β was 0.05237 (95% CI: 0.04372–0.06103), α/β = 3.78 Gy, *R*^2^ = 0.9949. The fitting equation was $\ln (s)=-0.20\cdotp d-0.05\cdotp{d}^2$. The survival fractions of 4 Gy/F × 2F and 6 Gy/F × 2F of fractioned radiation were corresponding to the curve and obtained corresponding single dose d_4 × 2_ = 7.93 Gy (actual value) and d_6 × 2_ = 10.83 Gy (actual value). At the same time, the α/β = 3.78 Gy was substituted into the BED formula and obtained equivalent single dose d_4 × 2_ = 6.22 Gy (calculated value) and d_6 × 2_ = 9.11 Gy (calculated value).

As shown in Table 1, for PC3 cells, when the α/β ratio was calculated according to 1.75 Gy, fractionated radiation was converted into the equivalent single high dose radiation with the conventional BED formula, and the calculated value was different from the actual measured value; the lowest calculated value was only 83% of the actual value. When 4 Gy/F× 2F was converted into an equivalent single dose, the actual value was 7.19 Gy, but after BED formula calculation, it was believed that only 5.96 Gy can satisfy clinical needs, which overestimated the ability of single hypofractionated radiation. If it was considered that the α/β ratio increased after single high dose radiation according to our previous research results, then substitute α/β ratio 3.46 and 4.24 Gy into the BED formula, respectively, Table 2 can be obtained [6].

**Table 1:** Differences between the calculated values and measured values of PC3 and DU145 cells when converted the fractionated irradiation dose into equivalent single irradiation dose

		Equivalent single dose (Gy)		
Cell lines	Fractionated mode	Calculated value (C)	Measured value (M)	C/M (%)
PC3	4 Gy/F × 2F	5.96	7.19	83
	6 Gy/F × 2F	8.81	9.16	96
DU145	4 Gy/F × 2F	6.22	7.93	78
	6 Gy/F × 2F	9.11	10.83	84

**Table 2:** Conversion with different α/β values in PC3 cells

Equivalent single dose (Gy)								
Cell lines	Fractionated mode	Calculated value (C)			Measured value (M)	C/M (%)		
		α/β = 1.75	α/β = 3.46	α/β = 4.24		α/β = 1.75	α/β = 3.46	α/β = 4.24
PC3	4 Gy/F × 2F	5.96	6.19	6.27	7.19	83	86	87
	6 Gy/F × 2F	8.81	9.06	9.17	9.16	96	99	100

Similarly, for DU145 cells, the α/β ratio was larger ~3.78 Gy. When the fractionated radiation was converted to the equivalent single high dose radiation, the difference between the calculated value and the actual measured value was larger when the BED formula was used, and the lowest value was only 78% of the actual value. When 4 Gy/F× 2F is converted into the equivalent single dose, the actual value was 7.93 Gy, but after BED formula calculation, it was also believed that only 6.22 Gy can satisfy clinical needs, and the same conclusion can be drawn. After substituting into the BED formula for recalculating with 6 and 10 Gy of the α/β ratio, respectively, Table 3 can be obtained.

**Table 3:** Conversion with different α/β values in DU145 cells

Equivalent single dose (Gy)								
Cell lines	Fractionated mode	Calculated value (C)			Measured value (M)	C/M (%)		
		α/β = 3.78	α/β = 6	α/β = 10		α/β = 3.78	α/β = 6	α/β = 10
DU145	4 Gy/F × 2F	6.22	6.43	6.70	7.93	78	81	84
	6 Gy/F × 2F	9.11	9.37	9.73	10.83	84	87	90

It can be seen from Tables 2 and 3 that when a fractionated radiation dose was converted into a single high radiation dose with the conventional BED formula, and the calculated value was closer to the actual value if a higher α/β ratio was used.

### The irreparable damage of single high dose radiation was more than that of fractionated radiation

**Fig. 2:**
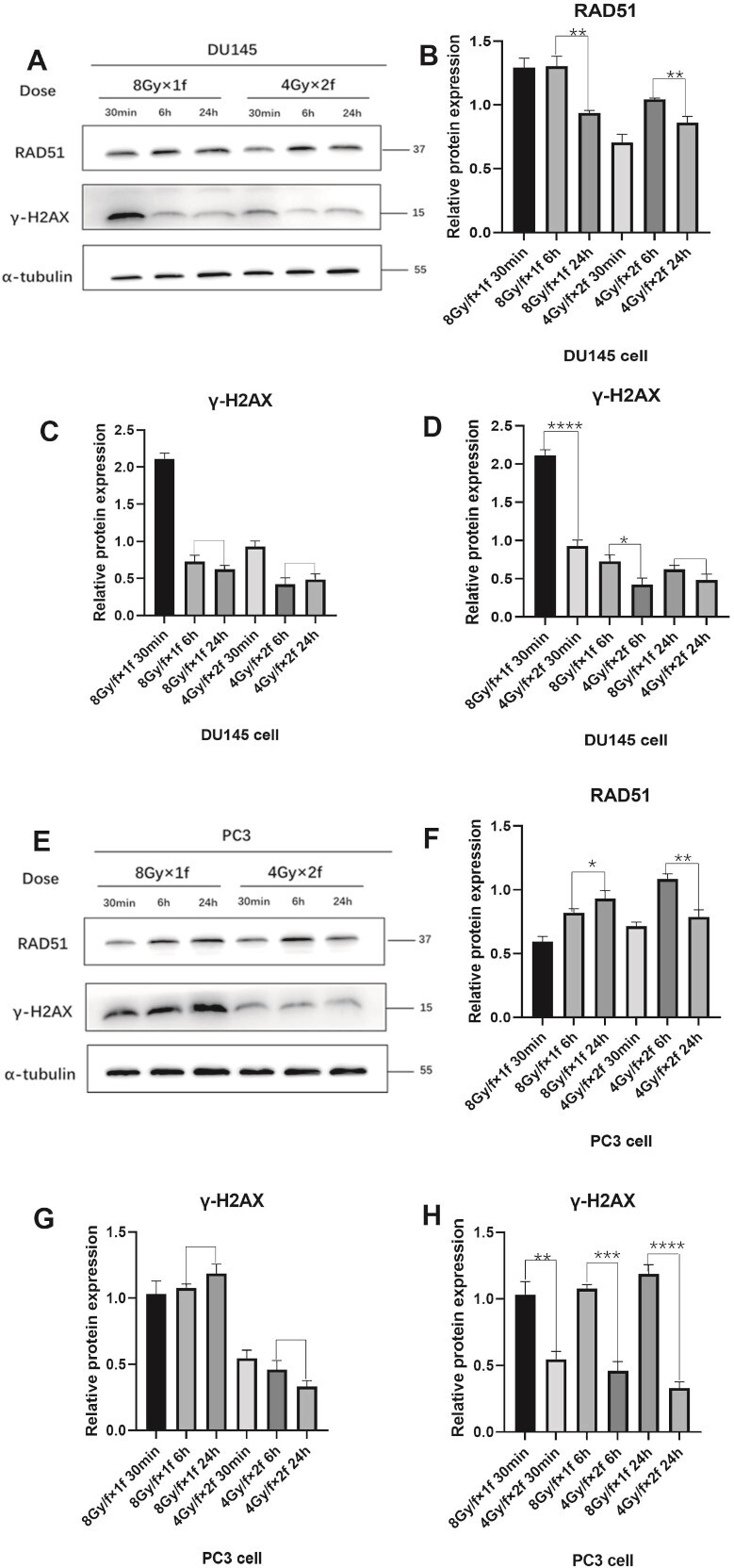
Western blot results of RAD51 and γ-H2AX in DU145 cells (Fig. 2A–D) and PC3 cells (Fig. 2E–H). Protein expression of RAD51 and γ-H2AX in DU145 cells (Fig. 2A) and PC3 cells (Fig. 2E) after irradiation in different fractionated modes (8 Gy/F × 1F and 4 Gy/F × 2F) at 30 min, 6 h and 24 h. Protein statistics (Fig. 2B–D and F–H). *, *P <* 0.05; **, *P <* 0.01; ***, *P <* 0.001; ****, *P <* 0.0001.

The western blot results were shown in Fig. 2A–D and E–H. At 30 min, 6 h and 24 h after a single high dose radiation of 8 Gy/F, the expression of damage marker γ-H2AX was higher than the fractionated radiation of 4 Gy/F × 2F (Fig. 2D and H). The expression of damage marker γ-H2AX was approximately the same after 6 and 24 h radiation in the two cell lines (Fig. 2C and G). The expression of repair protein RAD51 gradually increased after 30 min of radiation and was continuously expressed until 24 h (Fig. 2B and F). While the expression of damage marker γ-H2AX was not decreased, indicating that the existing DNA damage was not repaired. According to Hamasaki *et al.*, most of the damage at this time was irreparable damage [15].

**Fig. 3:**
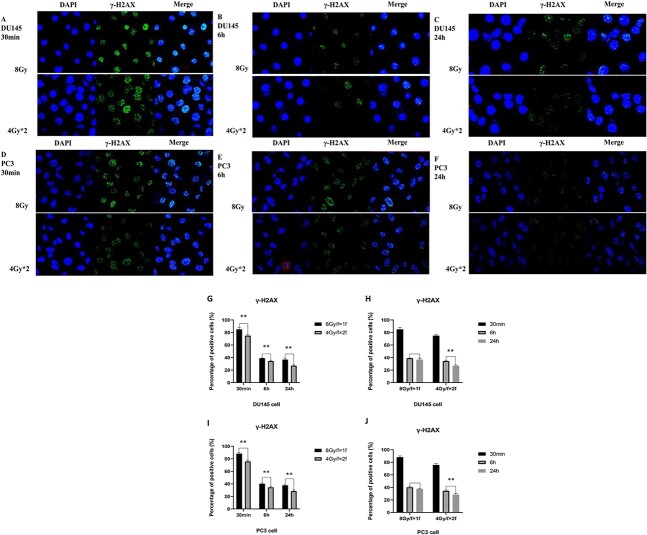
Fluorescence expression of γ-H2AX positive cells percentage in DU145 cells and PC3 cells. Fluorescence expression of γ-H2AX positive cells percentage in DU145 cells and PC3 cells after irradiation in different fractionated modes (8 Gy/F × 1F and 4 Gy/F × 2F) at 30 min, 6 h and 24 h were as follows: DU145 at 30 min (Fig. 3A), 6 h (Fig. 3B) and 24 h (Fig. 3C); PC3 30 min (Fig. 3D), 6 h (Fig. 3E), 24 h (Fig. 3F). Percentage of positive cells statistics (Fig. 3G–J). ^*^P < 0.05; ^*^^*^P < 0.01; ^*^^*^^*^P < 0.001.

As the expression of γ-H2AX of single high dose radiation was more than that of fractionated radiation after 6 h, the irreparable damage of single high dose radiation was also more than that of fractionated radiation. According to the definition of α/β ratio, α value represents irreparable damage, that is, when α value increased, α/β ratio increased.

### Some repairable damage may also be converted into irreparable damage after single hypofractionated radiation

As shown in Fig. 3A–J, the expression of γ-H2AX was gradually increased in the nucleus after radiation and then decreased with the process of damage repair. For different fractionated modes, the expression of γ-H2AX in both DU145 cells and PC3 cells increased significantly after single high dose radiation compared with fractionated radiation after 30 min, 6 h and 24 h, indicating more DNA damage (Fig. 3G and I).

In order to verify the damage repair after 6 h, the expression of γ-H2AX was also detected 24 h after radiation, and the results were shown in Fig. 3C and F. The expression of γ-H2AX was also different 24 h after radiation under different fractionated modes: 24 h after single high dose radiation (8 Gy/F × 1F), the expression of γ-H2AX was similar to that of 6 h. However, 24 h after fractionated radiation (4 Gy/F× 2F), the expression of γ-H2AX decreased slightly because there was still a small part of damage repair (Fig. 3H and J).

**Fig. 4:**
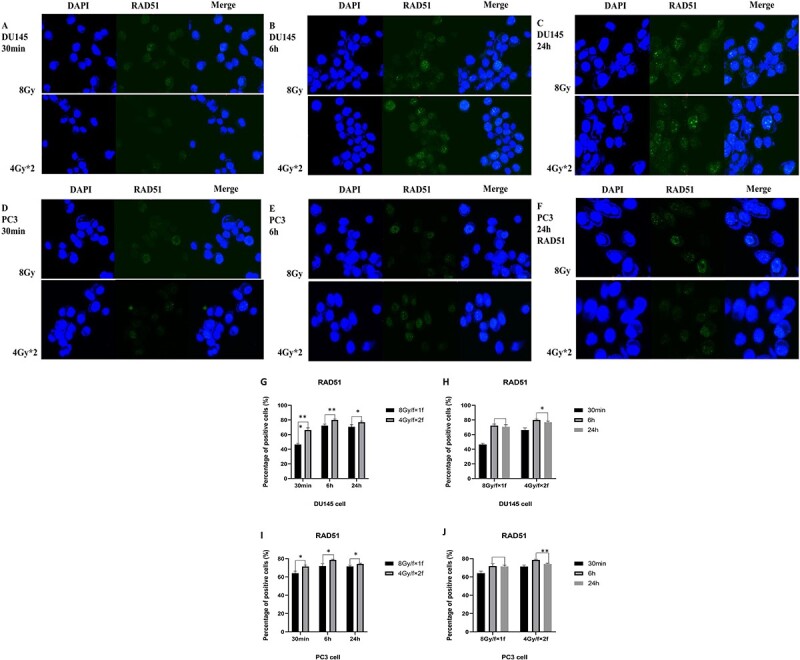
Fluorescence expression of RAD51 positive cells percentage in DU145 cells and PC3 cells. Fluorescence expression of RAD51 positive cells percentage in DU145 cells and PC3 cells after irradiation in different fractionated modes (8 Gy/F × 1F and 4 Gy/F × 2F) at 30 min, 6 h and 24 h were as follows: DU145 at 30 min (Fig. 4A), 6 h (Fig. 4B) and 24 h (Fig. 4C); PC3 30 min (Fig. 4D), 6 h (Fig. 4E), 24 h (Fig. 4F). Percentage of positive cells statistics (Fig. 4G–J). ^*^P < 0.05; ^*^^*^P < 0.01; ^*^^*^^*^P < 0.001.

As shown in Fig. 4A–J, the expression of RAD51 gradually increased with the appearance of DNA damage after radiation. For different fractionated modes, after 6 and 24 h, the expression of RAD51 with fractionated radiation was more than that of single high dose radiation (Fig. 4G and I). After 24 h, the expression of RAD51 irradiated by fractionated radiation was slightly lower than that after 6 h due to the completion of repair, while the expression of RAD51 after single high dose radiation was basically the same as that after 6 h (Fig. 4H and J).

Therefore, for a single high dose radiation, the repair protein RAD51 continued to express after 6 h, but the damage marker γ-H2AX was not reduced, indicating that the process of damage had been finished and the residual damage was irreparable damage. Meanwhile, some repairable damage may also be converted into irreparable damage. According to the definition of α/β ratio, α value represented irreparable damage, that is, α value increased. Some repairable SLD (β value) was converted into irreparable damage (α value), and the ratio increased when α value increased and β value decreased.

### When the dose per fraction increased, may need more fractions than calculated from conventional BED formula in murine xenograft model

**Fig. 5:**
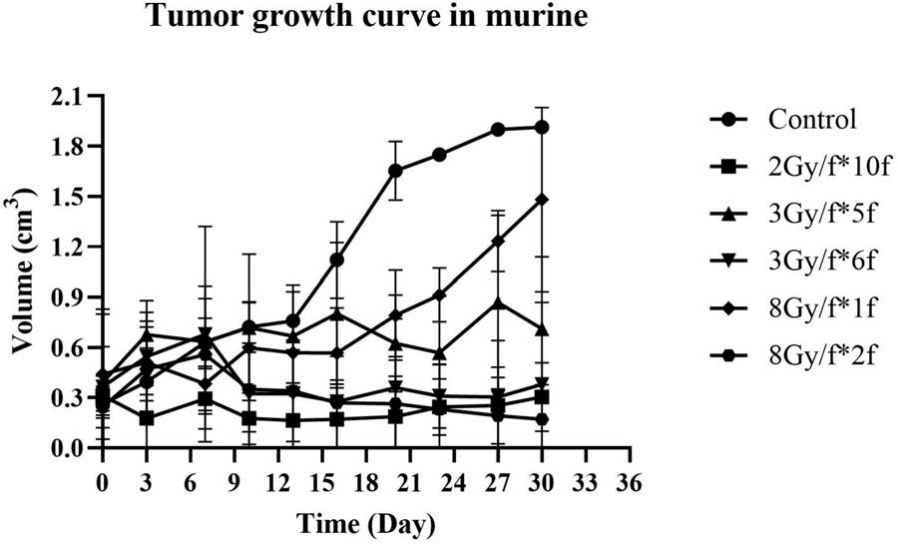
Tumor growth curve in PC3 cells on murine after different fractionated doses. We used the days after different fractionated radiation (control, 2 Gy/f × 10f, 3 Gy/f × 5f, 3 Gy/f × 6f, 8 Gy/f × 1f and 8 Gy/f × 2f) as the *X*-axis, the tumor volume as the *Y*-axis to draw the tumor growth curve of PC3 cells.

We established six groups according to different fractionated doses: control, 2 Gy/f × 10f, 3 Gy/f × 5f, 3 Gy/f × 6f, 8 Gy/f × 1f and 8 Gy/f × 2 f. When the transplanted tumor’s short diameter reached 0.5 cm, the murine were randomly assigned to each group. After being irradiated by different fractionated doses, tumor volumes were measured every 3 days. When the tumor volume reached 2 cm^3^, the murine were sacrificed. The tumor growth curve in murine was shown in Fig. 5.

The tumor growth curve reflected the actual biological effect of radiotherapy. As shown in Fig. 5, after different fractionated irradiation, the tumor growth curve of 2 Gy/f × 10f was nearly the same as 3 Gy/f × 6f and 8 Gy/f × 2 f. The significant difference test in different fractionated doses was shown in Table 4**.** However, generally, we consider the α/β ratio of prostate cancer tissue to be constant in the conventional notion, so when we substituted α/β ratio = 1.75 Gy into BED and equivalent dose in 2 Gy (EQD2) formula according to our results from PC3 cells, as shown in Table 5, the BED and EQD2 of 2 Gy/f × 10f were only near the same as 3 Gy/f × 5f and 8 Gy/f × 1f, less than the actual number of fractions. When we substituted α/β ratio = 3.46 Gy for 3 Gy/f and 4.24 Gy for 8 Gy/f according to our previous study to recalculate BED and EQD2, as shown in Table 5, the BED and EQD2 of 2 Gy/f × 10f were closer to 3 Gy/f × 6f and 8 Gy/f × 2f, the same result as it from our murine xenograft model. In general, when the dose per fraction increased, especially in an ultra-hypofractionated dose, a higher α/β ratio should be used and may need more fractions than calculated from the conventional BED formula.

**Table 4:** Significant difference test in different fractionated doses

ANOVA table	Sum of squares (Type III)	DF	Mean Square	*F*	*P* value
2 Gy/F × 10F vs. 3 Gy/F × 5F	2.620	1	2.620	35.01	<0.0001
2 Gy/F × 10F vs 3 Gy/F × 6F	0.3191	1	0.3191	3.756	**0.0612**
2 Gy/F × 10F vs 8 Gy/F × 1F	4.064	1	4.064	60.94	<0.0001
2 Gy/F × 10F vs 8 Gy/F × 2F	0.09769	1	0.09769	1.825	**0.1844**

**Table 5:** BEDs and EQD2s with different α/β ratios after different fractionated doses

Fractionated mode	α/β = 1.75 (Gy)		α/β = 3.46 (Gy)		α/β = 4.24 (Gy)	
	BED (Gy)	EQD2 (Gy)	BED (Gy)	EQD2 (Gy)	BED (Gy)	EQD2 (Gy)
2 Gy/F × 10F	**42.9**	**20**				
3 Gy/F × 5F	**40.7**	**19**	28	17.7		
3 Gy/F × 6F	48.9	22.8	**33.6**	**21.3**		
8 Gy/F × 1F	**44.6**	**20.8**			23.1	15.7
8 Gy/F × 2F	89.1	41.6			**46.2**	**31.4**

## DISCUSSION

With the rapid development of linear accelerators and the concept of precision radiotherapy being concerned by more and more doctors, conventional fractionated radiotherapy (2 Gy per fraction) has been far from satisfying clinical needs. Professor Zhang’s team published a landmark study in the Lancet in 2015: patients with operable stage I non-small cell lung cancer (NSCLC) who received stereotactic ablative radiotherapy (SABR) (54 Gy/3F or 50 Gy/4F) had a 3-year overall survival rate of 95%, much higher than 79% after surgery, further establishing the clinical position of hypofractionated radiotherapy [16]. Due to its radiobiology advantages, shortening treatment time and saving hospital and patient costs, the hypofractionated radiotherapy is increasingly favored by clinical doctors. However, with the popularization of hypofractionated radiotherapy in clinical work, more and more problems are emerging. A number of *in vitro* and *in vivo* studies have shown that the BED formula may not be applicable, which was used for bioequivalent conversion from conventional fractionated radiotherapy to hypofractionated radiotherapy [13, 17]. Moreover, in clinical practice, hypofractionated radiotherapy for prostate cancer patients did not obtain satisfactory results and only acquired that was not inferior to conventional fractionated radiotherapy [18–20]. We speculated that the α/β ratio derived from the LQ model may not be a constant, and our previous study using the maximum likelihood principle in mathematical statistics and clinical data confirmed that the α/β ratio of prostate cancer tends to become higher when the dose per fraction increases [6]. Therefore, this study verified this conclusion *in vitro* and *in vivo* to explore its potential mechanism.

There were many factors that affected α/β ratios, whether internal or external factors of cells, whether physical or chemical factors, and ultimately, it influenced the repairment of DNA damage caused by irradiation [5, 21]. Therefore, DNA repair after damage was correlated with the α/β ratio. From the definition of α/β ratio, α value represented irreparable damage and β value represented repairable SLD. If irreparable damage increased after single high dose irradiation, that is, α value increased; or part of repairable damage was converted into irreparable damage and β value decreased (α value increased), then the ratio increased.

H2AX was the most faithful variant of histone H2A. When DNA damage occurred, H2AX entered the center of cell reaction to DNA damage and phosphorylated near DSB to produce γ-H2AX. The number of γ-H2AX foci can roughly reflect the number of DSB and was consistent with DSB from the beginning of irradiation. After a period of time, due to DNA repair, the numbers of γ-H2AX foci reduced and the expression level decreased [8, 9, 22]. Therefore, observing and counting γ-H2AX foci in irradiated cells was a widely used method to verify the presence of DSB in DNA and to analyze its repairment [23].γ-H2AX foci can be detected only after a few minutes of irradiation and reached its maximum value after 30 min ~ 1 h, then gradually decreased [7]. By 6 h, the expression level of γ-H2AX was basically constant due to the completion of SLD, and most DSB detected at this time were irreparable damage [13, 15]. Therefore, it can be considered that the damage after 6 h was irreparable and represented the α value.

RAD51 was a recombinant enzyme that binds to BRCA2 to form presynaptic RAD51-BRCA2 nucleoprotein filaments on DNA. RAD51 was removed after sister chromatid invasion and homologous DNA sequence rearrangement, leaving a free 3′-OH terminal and enabling DNA polymerase to repair DNA in the 3′-5′ direction. Once the repaired DNA was completed, these enzymes broke down the Holliday junction, and the DNA ends were joined by DNA ligase I, completing the process of HR. Therefore, RAD51 was a key protein of HR [9, 24–26].

In conclusion, the number of γ-H2AX and DSB had a linear relationship. The expression of γ-H2AX increased with the increasing number of DSB after irradiation and decreased with the completion of DNA repair, so it can be used as a marker of DNA damage. RAD51, as the most important repair protein in HR pathway, can be used as a marker of DNA repair. At low doses and LETs, non-homologically-repairable damage (NHEJ) was predominant with high fidelity [27, 28]. However, at single high dose radiation and LETs, NHEJ was inaccurate and required intervention of the high-fidelity HR repair system [29]. In this study, the expression levels of DNA damage marker γ-H2AX and DNA repair marker RAD51 were changed after radiation with different fractionated modes, so as to verify the influence of different fractionated modes on DNA damage and repair and to prove the change of α/β ratio after hypofractionated radiotherapy from the definition of α/β ratio.

The results of this study showed that the expression of DNA damage marker γ-H2AX was higher after 6 h with single high dose radiation than fractionated radiation, and the difference lasted 24 h, indicating that single high dose radiation had more DNA damage. After 6 h, the DNA repair marker RAD51 continued to be highly expressed until 24 h after single high dose radiation. DNA damage still existed even with the expression of repair protein, meaning the damage was irreparable. In conclusion, irreparable damage after single high dose radiation was more than that of fractionated radiation. At the same time, according to the results of immunofluorescence, the expression of γ-H2AX decreased after 24 h compared with 6 h after fractionated radiation. But the expression of γ-H2AX after 24 h showed no significant difference compared with 6 h after single high dose radiation, indicating that single high dose radiation can transform part of repairable damage into irreparable damage. According to the definition of α/β ratio, α value represented irreparable damage, that is, the α value increased; some repairable damage (β value) was converted to irreparable damage (α value), and the ratio increased when the α value increased and the β value decreased, which was consistent with the conclusion of our previous study, confirming the view that the α/β ratio increased after the single high dose irradiation [6].

In recent years, several models, especially revised LQ models that take DNA damage repair and time factor into consideration, have been proposed for evaluating fractionated irradiation and single high dose irradiation. For the time-dependent model, time factor was considered, and the proportion of repairable damages after high-LET irradiation (0.1–0.15) was much lower than low-LET irradiation (0.9); Meanwhile, after high-LET irradiation, the proportion of ‘a single hit’ that results in lethal damage can be expressed approximately by a large value (0.9). The implication is a decrease in repairable damage and an increase in irreparable damage after high-LET irradiation. And high-LET irradiation could be regarded as a special type of single high dose irradiation. Therefore, it is consistent with our conclusion [30]. For the universal survival curve (USC) model, combined the LQ model (for low dose) and multitarget model (for high dose) [31]. Linear-quadratic-linear (LQL) model based on lethal-potentially lethal theory was more complex to calculate [32]. Both USC model and LQL model cell survival curves extend nearly linearly in a high-dose meaning α factor (linear coefficient) was predominant [33]. The conclusions of the two new models were consistent with our views. For the generalized LQ (gLQ) model, it took SLD and lethal damage into account, and they thought that SLD was converted to lethal damage during high dose irradiation, which is in line same with our opinion [34]. In conclusion, for the above modified model, the revised formulas were complex and cumbersome to apply and may not be suitable for clinical use. In our viewpoint, we just need to substitute a larger α/β ratio without modifying the familiar LQ model.

In this study, *in vitro* and *in vivo* experiments demonstrated that the α/β ratio increased after hypofractionated radiotherapy, especially in ultra-hypofractionated doses, and may need more fractions than calculated from the conventional BED formula to balance the overestimation caused by the high dose irradiation.

## References

[1] Ghate A . Imputing radiobiological parameters of the linear-quadratic dose-response model from a radiotherapy fractionation plan. Phys Med Biol 2020;65:225009. 10.1088/1361-6560/abb935. 32937610

[2] Withers H . Biologic basis for altered fractionation schemes. Cancer 1985;55:2086–95. 10.1002/1097-0142(19850501)55:9+<2086::aid-cncr2820551409>3.0.co;2-1. 3919923

[3] Fowler JF . The linear-quadratic formula and progress in fractionated radiotherapy. Br J Radiol 1989;62:679–94. 10.1259/0007-1285-62-740-679. 2670032

[4] Hendry J, Bentzen S, Dale R et al. A modelled comparison of the effects of using different ways to compensate for missed treatment days in radiotherapy. Clin Oncol (R Coll Radiol) 1996;8:297–307. 10.1016/s0936-6555(05)80715-0. 8934049

[5] Barendsen GW, Van Bree C, Franken NA. Importance of cell proliferative state and potentially lethal damage repair on radiation effectiveness: implications for combined tumor treatments (review). Int J Oncol 2001;19:247–56. 10.3892/ijo.19.2.247. 11445835

[6] Cui M, Gao X, Li X et al. Variability of α/β ratios for prostate cancer with the fractionation schedule: caution against using the linear-quadratic model for hypofractionated radiotherapy. Radiat Oncol 2022;17:54. 10.1186/s13014-022-02010-9. 35303922 PMC8932192

[7] Kinner A, Wu W, Staudt C, Iliakis G. Gamma-H2AX in recognition and signaling of DNA double-strand breaks in the context of chromatin. Nucleic Acids Res 2008;36:5678–94. 10.1093/nar/gkn550. 18772227 PMC2553572

[8] Horn S, Barnard S, Rothkamm K. Gamma-H2AX-based dose estimation for whole and partial body radiation exposure. PLoS One 2011;6:e25113. 10.1371/journal.pone.0025113. 21966430 PMC3179476

[9] Borrego-Soto G, Ortiz-Lopez R, Rojas-Martinez A. Ionizing radiation-induced DNA injury and damage detection in patients with breast cancer. Genet Mol Biol 2015;38:420–32. 10.1590/S1415-475738420150019. 26692152 PMC4763322

[10] Sánchez H, Paul M, Grosbart M et al. Architectural plasticity of human BRCA2-RAD51 complexes in DNA break repair. Nucleic Acids Res 2017;45:4507–18. 10.1093/nar/gkx084. 28168276 PMC5416905

[11] Guha S, Bhaumik S. Transcription-coupled DNA double-strand break repair. DNA Repair 2022;109:103211. 10.1016/j.dnarep.2021.103211. 34883263

[12] Shibamoto Y, Ito M, Sugie C et al. Recovery from sublethal damage during intermittent exposures in cultured tumor cells: implications for dose modification in radiosurgery and IMRT. Int J Radiat Oncol Biol Phys 2004;59:1484–90. 10.1016/j.ijrobp.2004.04.039. 15275736

[13] Iwata H, Shibamoto Y, Murata R et al. Estimation of errors associated with use of linear-quadratic formalism for evaluation of biologic equivalence between single and hypofractionated radiation doses: an in vitro study. Int J Radiat Oncol Biol Phys 2009;75:482–8. 10.1016/j.ijrobp.2008.12.093. 19735872

[14] Thielhelm T, Nourbakhsh A, Welford S et al. RAD51 inhibitor and radiation toxicity in vestibular schwannoma. Otolaryngol Head Neck Surg 2022;167:860–8. 10.1177/01945998221083506. 35230908 PMC9433467

[15] Hamasaki K, Imai K, Nakachi K et al. Short-term culture and gammaH2AX flow cytometry determine differences in individual radiosensitivity in human peripheral T lymphocytes. Environ Mol Mutagen 2007;48:38–47. 10.1002/em.20273. 17163504

[16] Chang J, Senan S, Paul M et al. Stereotactic ablative radiotherapy versus lobectomy for operable stage I non-small-cell lung cancer: a pooled analysis of two randomised trials. Lancet Oncol 2015;16:630–7. 10.1016/s1470-2045(15)70168-3. 25981812 PMC4489408

[17] Otsuka S, Shibamoto Y, Iwata H et al. Compatibility of the linear-quadratic formalism and biologically effective dose concept to high-dose-per-fraction irradiation in a murine tumor. Int J Radiat Oncol Biol Phys 2011;81:1538–43. 10.1016/j.ijrobp.2011.05.034. 22115556

[18] Incrocci L, Wortel RC, Alemayehu WG et al. Hypofractionated versus conventionally fractionated radiotherapy for patients with localised prostate cancer (HYPRO): final efficacy results from a randomised, multicentre, open-label, phase 3 trial. Lancet Oncol 2016;17:1061–9. 10.1016/s1470-2045(16)30070-5. 27339116

[19] Lee WR, Dignam JJ, Amin MB et al. Randomized phase III noninferiority study comparing two radiotherapy fractionation schedules in patients with low-risk prostate cancer. J Clin Oncol 2016;34:2325–32. 10.1200/JCO.2016.67.0448. 27044935 PMC4981980

[20] Widmark A, Gunnlaugsson A, Beckman L et al. Ultra-hypofractionated versus conventionally fractionated radiotherapy for prostate cancer: 5-year outcomes of the HYPO-RT-PC randomised, non-inferiority, phase 3 trial. Lancet 2019;394:385–95. 10.1016/s0140-6736(19)31131-6. 31227373

[21] Franken NAP, Oei AL, Kok HP et al. Cell survival and radiosensitisation: modulation of the linear and quadratic parameters of the LQ model. Int J Oncol 2013;42:1501–15. 10.3892/ijo.2013.1857. 23503754

[22] Stenvall A, Larsson E, Holmqvist B et al. Quantitative γ-H2AX immunofluorescence method for DNA double-strand break analysis in testis and liver after intravenous administration of InCl. EJNMMI Res 2020;10:22. 10.1186/s13550-020-0604-8. 32189079 PMC7080928

[23] Keta O, Petković V, Cirrone P et al. DNA double-strand breaks in cancer cells as a function of proton linear energy transfer and its variation in time. Int J Radiat Biol 2021;97:1229–40. 10.1080/09553002.2021.1948140. 34187289

[24] Williams A, Michael W. Eviction notice: new insights into Rad51 removal from DNA during homologous recombination. Mol Cell 2010;37:157–8. 10.1016/j.molcel.2010.01.009. 20122398

[25] Matos J, West S. Holliday junction resolution: regulation in space and time. DNA Repair 2014;19:176–81. 10.1016/j.dnarep.2014.03.013. 24767945 PMC4065333

[26] Shahid T, Soroka J, Kong E et al. Structure and mechanism of action of the BRCA2 breast cancer tumor suppressor. Nat Struct Mol Biol 2014;21:962–8. 10.1038/nsmb.2899. 25282148 PMC4222816

[27] Liu M, Lee S, Liu B et al. Ku-dependent non-homologous end-joining as the major pathway contributes to sublethal damage repair in mammalian cells. Int J Radiat Biol 2015;91:867–71. 10.3109/09553002.2015.1075178. 26189733 PMC4748373

[28] Matsumoto Y, Ando K, Kato TA et al. Difference in degree of sub-lethal damage recovery between clinical proton beams and x-rays. Radiat Prot Dosim 2019;183:93–7. 10.1093/rpd/ncy270. 30576477

[29] Brahme A . A DNA repair-based model of cell survival with important clinical consequences. Radiat Res 2020;194:202–35. 10.1667/RADE-20-00052.1. 32942300

[30] Sakae T, Takada K, Kamizawa S et al. Formulation of time-dependent cell survival with saturable repairability of radiation damage. Radiat Res 2023;200:139–50. 10.1667/RADE-21-00066.1. 37303133

[31] Park C, Papiez L, Zhang S et al. Universal survival curve and single fraction equivalent dose: useful tools in understanding potency of ablative radiotherapy. Int J Radiat Oncol Biol Phys 2008;70:847–52. 10.1016/j.ijrobp.2007.10.059. 18262098

[32] Guerrero M, Carlone M. Mechanistic formulation of a lineal-quadratic-linear (LQL) model: split-dose experiments and exponentially decaying sources. Med Phys 2010;37:4173–81. 10.1118/1.3456927. 20879577

[33] Shibamoto Y, Otsuka S, Iwata H et al. Radiobiological evaluation of the radiation dose as used in high-precision radiotherapy: effect of prolonged delivery time and applicability of the linear-quadratic model. J Radiat Res 2012;53:1–9. 10.1269/jrr.11095. 21997195

[34] Wang JZ, Huang Z, Lo SS et al. A generalized linear-quadratic model for radiosurgery, stereotactic body radiation therapy, and high-dose rate brachytherapy. Sci Transl Med 2010;2:39ra48. 10.1126/scitranslmed.3000864. 20610850

